# Anaphylaxis in an infant to raw potato

**DOI:** 10.1186/2045-7022-1-S1-P53

**Published:** 2011-08-12

**Authors:** Pinar Uysal, Zeynep Arikan Ayyildiz, Senol Alan, Tuba Tuncel, Fatih Firinci, Ozkan Karaman, Nevin Uzuner

**Affiliations:** 1 Dokuz Eylul University Medical Faculty, Department of Pediatrics Division of Allergy, Izmir, Turkey; 2 Zonguldak Karaelmas University Science and Art Faculty, Department of Biology, Zonguldak, Turkey

## Introduction

Potato was believed to have a lower allergenic potential and it was the one of the first preferred food for weaning period in infancy. To date, the allergenic reactions are mostly reported in adults including oral allergy syndrome, contact dermatitis, exacerbations of asthma, and rarely anaphylaxis.

## Case

A 10 months-old male infant admitted to emergency department with a history of general hyperemic itchy rash, swelling around his eyes and lips, vomiting, respiratory difficulty and wheezing which were started a few minutes later after the first contact with raw potato through his hands and face while playing with it. He was diagnosed as anaphylaxis and administered epinephrine IM, nebulised salbutamol, systemic antihistaminine and corticosteroid. His previous history was normal about atopy and he had eaten cooked potato for several times before. Spesific IgE level was 7.2 kU/L by ImmunoCAP (Phaida, Uppsala, Sweden) for potato. Prick-to-prick tests were performed and they were 9 mm for histamine, 13 mm for raw potato, although they were negative to cooked potato, apple and pear which might have a high probability for cross-reaction with potato. Skin prick tests with commercial extracts of latex, birch pollen, mixture of grasses, trees, cereals and weeds were all negative, as well. Immunoblot of raw and cooked potato extract were performed. Protein staining after SDS-PAGE showed different bands in the range of 5 to >90 kDa for raw potato. Immunostaining revealed a distinct IgE binding band around 92.7 kDa area in raw potato and no reaction was detected for cooked potato. A strict elimination diet for potato, label reading, and epinephrine injection were recommended.

## Conclusion

To the best of our knowledge, this case is the smallest reported infant who had an anaphylactic reaction to raw potato at his first exposure. That novel protein might be the causative allergenic protein for raw potato allergy and moreover it is most probably a heat-labile protein.

